# Clinicopathologic Features of IgG4-Related Kidney Disease

**DOI:** 10.1016/j.ekir.2024.05.011

**Published:** 2024-05-15

**Authors:** Alessia Buglioni, Sarah M. Jenkins, Samih H. Nasr, Pingchuan Zhang, Ian W. Gibson, Mariam P. Alexander, Loren P. Herrera Hernandez, Mary E. Fidler, Naoki Takahashi, Marie C. Hogan, Lynn D. Cornell

**Affiliations:** 1 Division of Anatomic Pathology, Department of Laboratory Medicine and Pathology, Mayo Clinic, Rochester, Minnesota, USA; 2 Division of Clinical Trials and Biostatistics, Mayo Clinic, Rochester, Minnesota, USA; 3 Department of Pathology, University of Manitoba College of Medicine, Winnipeg, Manitoba, Canada; 4 Division of Abdominal Imaging, Department of Radiology, Mayo Clinic, Rochester, Minnesota, USA; 5 Division of Nephrology and Hypertension, Department of Medicine, Mayo Clinic, Minnesota, USA

**Keywords:** autoimmune, IgG4-related disease, interstitial nephritis, membranous nephropathy, tubulointerstitial nephritis

## Abstract

**Introduction:**

IgG4-related disease (IgG4-RD) is a systemic immune-mediated disease that can involve nearly any organ. IgG4-RD can affect the kidney in different disease patterns, collectively referred to as IgG4-related kidney disease (IgG4-RKD).

**Methods:**

We conducted a tissue-based cohort study with clinicopathological correlation in 125 patients with IgG4-RKD.

**Results:**

The mean age at biopsy (*n* = 120) or nephrectomy (*n* = 5) was 63 years; 80% were male. One hundred eighteen patients (94%) had IgG4-related tubulointerstitial nephritis (IgG4-TIN); 20 patients (16%) had IgG4-related membranous glomerulonephritis (IgG4-MGN; 13 with concurrent IgG4-TIN). The primary clinical indication for biopsy/nephrectomy was acute or chronic renal failure in 78%, proteinuria in 17%, and mass lesion(s) in 15% (with overlap in primary indication). Fifty-two percent patients (41/79) had abnormal radiographic findings, including masses in 30% (24/79). All patients with IgG4-MGN had proteinuria. Extrarenal involvement by IgG4-RD was present in 79%. Median serum creatinine at presentation was 2.5 mg/dl (range 0.7–12). Serum IgG and/or IgG4 was increased in 91% (53/58); hypocomplementemia was present in 56% (43/77). Light microscopy showed plasma cell–rich interstitial nephritis in all cases of IgG4-TIN. Ninety-two percent of patients showed increased IgG4+ plasma cells. Seven percent showed an acute interstitial nephritis (AIN) pattern, and 5% showed non-necrotizing arteritis. Tubular basement membrane immune deposits were present in 83% of IgG4-TIN. Treatment information was available for 71 patients; 62 were treated with immunosuppression. Of those with elevated creatinine, 72% (41/57) showed a treatment response.

**Conclusion:**

This largest tissue-based series more clearly defines the disease phenotype of IgG4-RKD.

## Graphical abstract

**Figure:**
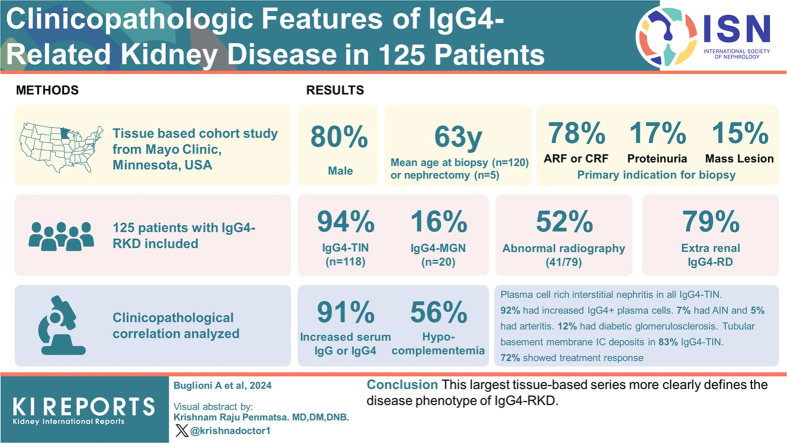

IgG4-RD is a systemic immune-mediated process that can affect virtually any organ system. Patients can present with inflammatory masses, functional organ impairment, or both.^1^, ^2^, ^3^, ^4^ In the case of a mass lesion, the clinical suspicion includes malignancy. Patients may also present with organ functional impairment through symptoms and/or laboratory-detected organ dysfunction (e.g., dry mouth/eye or increased serum creatinine) which would lead to further investigation, including biopsy of the affected tissue.

Kidney involvement is referred to as IgG4-RKD, which is not a single specific disease pattern but includes IgG4-TIN, glomerular disease (most commonly IgG4-MGN), rarely IgG4 plasma cell arteritis, and IgG4-related pyelitis.^5^, ^6^, ^7^, ^8^, ^9^, ^10^, ^11^ The kidney may also be secondarily affected by obstruction due to retroperitoneal fibrosis and periureteral lesions due to IgG4-RD.^12^^,^^13^

Previous tissue-based studies highlighted the clinicopathological characteristics of IgG4-RKD in Japanese and North American cohorts, including 23 and 35 patients, respectively.^9^^,^^10^ In the Japanese study, clinical and therapeutic follow-up information was also more recently gathered.^14^ A recent study from China of IgG4-RKD included 42 patients.^15^ A more recent work by Evans *et al.*^16^ studied 154 subjects affected by IgG4-RD in the United Kingdom. Among these, 14 patients (9%) had renal involvement. Similarly, in the Wallace *et al.*^1^ study of 125 IgG4-RD patients, 15 (12%) had renal involvement. A recent clinical study from France and Belgium included 101 patients with IgG4-RKD, some of whom had biopsies, and all of whom showed IgG4-TIN +/− MGN.^17^

The goal of our study is to enrich the current clinicopathological knowledge of IgG4-RKD, with a particular emphasis on the histopathologic and immunophenotypic features and clinical and laboratory correlates, in this largest tissue-based series of IgG4-RKD.

## Methods

This study was approved by the Mayo Clinic Institutional Review Board. A retrospective analysis with clinicopathological correlation was performed in 125 patients consecutively diagnosed with IgG4-RKD between January 2001 and April 2023. Biopsies and nephrectomies were obtained from patients who underwent biopsy or nephrectomy at Mayo Clinic, from patients who underwent medical renal biopsy outside of Mayo Clinic and had tissue sent to Mayo Clinic for processing, and a small number of consult cases initially interpreted by pathologists elsewhere and reviewed at the Mayo Clinic in Rochester, Minnesota. Twenty-seven patients included in this study were previously reported as part of 2 previous series and 1 case report.^7^^,^^10^^,^^18^ To qualify as IgG4-TIN, renal specimens must have shown a plasma cell–rich tubulointerstitial nephritis (TIN) pattern and at least 1 of the following criteria: (i) clinical evidence of other organ involvement by IgG4-RD, (ii) laboratory results of increased serum total IgG or IgG4 levels or hypergammaglobulinemia, or (iii) radiographic features in the kidney of small peripheral cortical nodules, round or wedge-shaped lesions, or diffuse patchy involvement by computed tomography scan or magnetic resonance imaging as described by Takahashi *et al.*^12^ or marked diffuse enlargement of kidneys by ultrasound. In addition, patients with other diseases mimicking IgG4-TIN clinically and histopathologically (e.g., ANCA associated interstitial inflammation and chronic pyelonephritis) were excluded. To qualify as IgG4-MGN, renal biopsies must have shown a membranous glomerulonephritis pattern of injury and concurrent IgG4-TIN or other organ involvement by IgG4-RD. For patients with biopsies showing features of IgG4-TIN with few IgG-positive plasma cells (≤10/40 × high power microscopic field [HPF]), the diagnosis of IgG4-TIN was made by the presence of a typical histologic pattern on biopsy in conjunction with the clinical history (in particular other organ involvement by IgG4-RD) and exclusion of mimickers. Presentation and medical history, imaging, laboratory findings, treatment, and follow-up were obtained from referral forms submitted at the time of biopsy and patients’ medical records. Laboratory data included serum creatinine, serum protein electrophoresis, serum total IgG and IgG4, presence of antinuclear antibodies (ANA) and anti-dsDNA, antineutrophil cytoplasmic antibodies (ANCA, anti-myeloperoxidase and anti-proteinase 3), serum complement (C3 and C4), hepatitis B and C results, hematuria, and proteinuria. Among those with elevated creatinine at the time of biopsy/nephrectomy, we defined treatment response as a decrease of at least 0.3 mg/dl in creatinine. Patients with end-stage kidney disease (ESKD) or dialysis on follow-up were included among treatment nonresponders.

At our institution, standard processing of renal biopsies includes light microscopy, immunofluorescence (IF) and electron microscopy (EM). For light microscopy, all cases were stained with hematoxylin and eosin, periodic acid Schiff, Masson’s trichrome and Jones methenamine silver. The degree of cortical interstitial fibrosis and tubular atrophy was defined as mild (10–25%), moderate (26–50%), or severe (>50%).

On sections from paraffin-embedded tissue, immunohistochemistry was performed with a mouse monoclonal antibody to human IgG4 (Cell Marque, Rocklin, CA) and a polyclonal rabbit antihuman IgG (Dako, Denmark). IgG4-positive plasma cells were counted per 400× magnification (40× microscopic field, HPF) or 0.22 mm^2^ circle on a scanned slide, and graded in the single most concentrated area on a biopsy as follows: no increase <5 cells/HPF, mild 5 to 10 cells/HPF, moderate 11 to 30 cells/HPF, and marked >30 cells/HPF.^10^^,^^19^ IgG-positive plasma cells were counted in the same area to obtain an IgG4/IgG plasma cell ratio.^20^

For IF, 3 μm cryostat sections were stained with polyclonal fluorescein isothiocyanate–conjugated antibodies to IgG, IgM, IgA, C3, C1q, kappa, lambda, fibrinogen, and albumin (Dako Corp, Denmark). IgG subclasses were tested using mouse monoclonal fluorescein isothiocyanate–conjugated antibodies to IgG1, IgG2, IgG3, and IgG4 (Sigma-Aldrich, Israel). Additional testing by IF for phospholipase A2 receptor (PLA2R) and thrombospondin type-1 domain-containing 7A was performed in cases of MGN. IF was scored on a 0 to 3+ scale. In IgG4-TIN specimens without material originally submitted for EM, EM was performed on deparaffinized tissue obtained from the light microscopy blocks, if tissue was available.

Patient characteristics were summarized with frequencies and percentages or with means, SD, medians, or ranges, as appropriate. Continuous variables were compared between groups of interest (i.e., TIN only vs. MGN [+/− TIN]) with Wilcoxon rank-sum tests, and categorical variables were compared with Fisher’s exact test. *P*-values ≤ 0.05 were considered statistically significant. All analyses were performed using SAS version 9.4 (SAS Institute Inc., Cary, North Carolina).

## Results

### Characteristics of the Entire Cohort

A total of 125 patients (100 male and 25 female) were identified with IgG4-RKD on biopsy (*n* = 120) or nephrectomy for mass (*n* = 5). The mean age at biopsy or nephrectomy was 63 years (range 20–84 years). All were native kidneys except for 1 case of recurrent IgG4-TIN in the kidney transplant (previously reported).^18^ IgG4-TIN accounted for approximately 1.3% of all native kidney biopsies that had a diagnosis of acute or chronic tubulointerstitial nephritis during the time of January through December 2022 (6 IgG4-TIN biopsies during this time, with 462 native kidney biopsies showing acute or chronic TIN of any cause).

**Figure 1:**
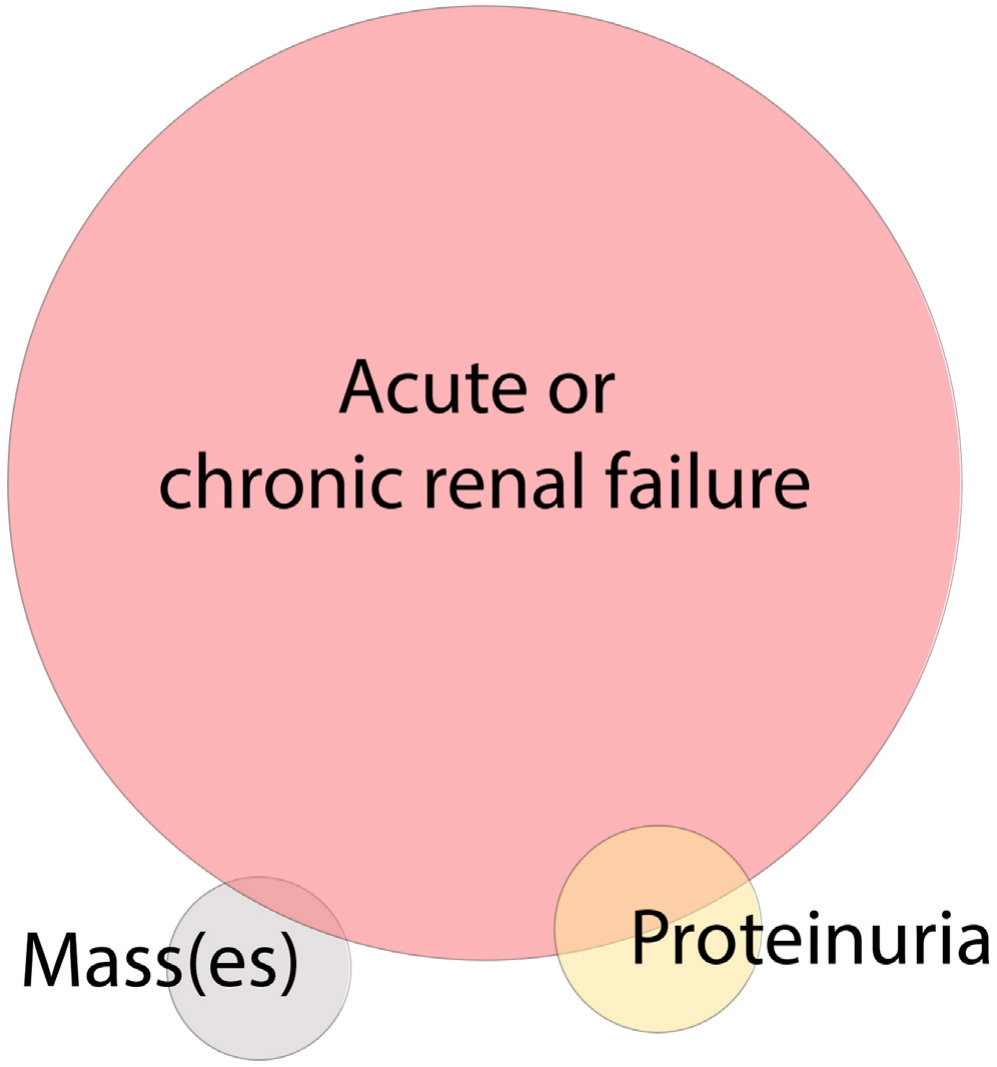
Primary indication for biopsy/nephrectomy in IgG4-RKD. Distribution of primary indication for diagnostic procedure in our cohort. Most patients underwent biopsy for acute and/or chronic renal failure, while a smaller percentage underwent biopsy/nephrectomy for mass lesion(s) or proteinuria, with some overlap in primary indication. The size of the circles and degree of overlap is proportional to the number of patients in each category.

The median serum creatinine at presentation was 2.5 mg/dl (range 0.7–12). At presentation, 14 patients (12%) had normal creatinine (defined as ≤1.2 mg/dl). Patients more likely to have normal creatinine were those with IgG4-MGN without TIN (83% had normal creatinine) and those who underwent biopsy or nephrectomy primarily for a mass lesion (57% had normal creatinine). Samples showed TIN in 94% (118/125); MGN, with or without accompanying TIN, was present in 16% (20/125). The primary indication for biopsy was acute kidney injury or chronic kidney disease in 78% (97/125), proteinuria in 17% (21/125), and mass lesion(s) in 15% (19/125); 13% of patients had overlapping primary indications for biopsy (Figure 1). Although not necessarily the primary indication for biopsy/nephrectomy, overall, 52% (41/79) had abnormal radiographic findings. There were similar rates of abnormal radiographic findings in the earlier versus later IgG4-RD “eras” (before 2012, and 2012 and later), at 51% and 55% respectively. Among the radiographic abnormalities identified, mass(es) were the most common finding, in 30% (24/79), followed by enlarged kidneys (13%), an infiltrative pattern (6%) and hydronephrosis (2%). The imaging modality was computerized tomography (CT) scan in 28 patients, ultrasound (+/− CT) in 10, and magnetic resonance scanning (MRI) (+/− CT) in 7. By ultrasound, 8 of 10 showed enlargement and 2 of 10 showed mass lesion(s); by CT or MRI, 24 of 33 showed mass(es) and 3 of 33 showed enlarged kidneys.

**Figure 2:**
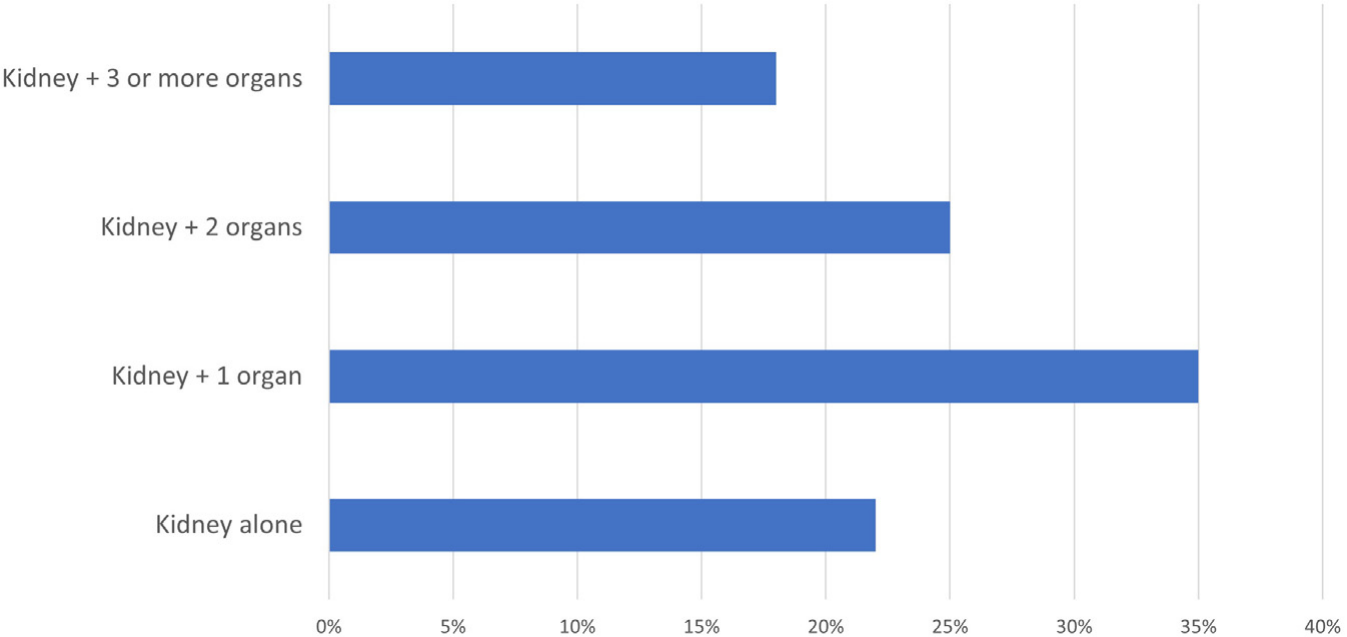
Multi-organ involvement in IgG4-RKD. Number of organs involved besides kidney in IgG4-RKD.

Extrarenal involvement was present in 79% of the cases with information available (75/95). Overall, 21% of patients had involvement of the kidney alone, 36% had 1 additional organ involved, 25% had 2 additional organs, and 18% had 3 or more (Figure 2). The most common other organ(s) or tissue(s) involved were the pancreas (40%) followed by liver/biliary tract (18%), salivary glands (17%), lung (16%), retroperitoneum (retroperitoneal fibrosis) (9%), skin (3%); thyroid, testis, aorta, sinus, eye/orbit, lacrimal gland (each 2%); and 1 each of heart (pericarditis), bladder, and stomach/duodenum. There was known lymphadenopathy in 26% of patients, although this is likely an underestimate as complete information was not available for all patients since lymphadenopathy was usually determined by chest or abdominal radiographic studies, which were not available in many patients.

Serum IgG and/or IgG4 was increased in 91% of patients (53/58) and serum IgG4 was increased (>135 mg/dl) in 81% (35/43). The median serum IgG4 was 493 mg/dl (range 94–3240 mg/dl; numeric data were available on only 28 patients). Peripheral blood eosinophilia was present in 43% (17/40) of patients with information available. Serologic studies for hepatitis B and C were negative in 98% of patients (52/53); 1 patient had a positive test for hepatitis B. C3 and/or C4 were decreased in 56% (43/77). Thirty percent (23/77) had a positive ANA and 2 patients (2%) had positive anti-dsDNA. One patient (1/71) had a low titer transiently positive p-ANCA and antimyeloperoxidase, in the setting of markedly elevated serum IgG4 levels, and TIN with tubular basement membrane (TBM) deposits, and without crescentic or necrotizing glomerular injury, and so this biopsy was considered to show IgG4-TIN rather than ANCA-associated disease. Clinical and laboratory findings are summarized in Table 1.

**Table 1:** Summary of clinical, laboratory, and histopathologic features in IgG4-RKD^a^

Characteristic	TIN only (*N* = 105)	MGN (+/− TIN) (*N* = 20)	Total (*N* = 125)	*P*-value
Gender				0.23
Female	19 (18.1%)	6 (30.0%)	25 (20.0%)	
Male	86 (81.9%)	14 (70.0%)	100 (80.0%)	
Age at biopsy/nephrectomy (yr)				0.17
Number of patients	105	20	125	
Mean (SD)	63.5 (12.8)	60.5 (10.8)	63.0 (12.5)	
Median	66.0	62.5	66.0	
Q1, Q3	59.0, 72.0	53.5, 67.5	58.0, 72.0	
Range	(20.0–84.0)	34–75	(20.0–84.0)	
Specimen type				
Biopsy	100 (95.2%)	20 (100.0%)	120 (96.0%)	1.0
Nephrectomy	5 (4.8%)	0 (0.0%)	5 (4.0%)	
Baseline creatinine (mg/dl)				0.21
Number of patients	94	19	113	
Mean (SD)	3.2 (2.0)	3.4 (3.3)	3.2 (2.2)	
Median	2.6	2.1	2.5	
Range	(0.8–10.0)	(0.7–12.0)	(0.7–12.0)	
≤1.2 (“normal”)	8 (8.5%)	6 (31.6%)	14 (12.4%)	0.01
>1.2 (high)	86 (91.5%)	13 (68.4%)	99 (87.6%)	
Proteinuria	42/76 (55.3%)	18/18 (100%)	60/94 (63.8%)	0.0002
Hematuria	17/75 (22.7%)	5/15 (33.3%)	22/90 (24.2%)	0.51
Proteinuria and hematuria	13/75 (17.3%)	5/15 (33.3%)	18/90 (20.0%)	0.17
Hypocomplementemia (low C3 or C4)	41/64 (64.1%)	2/13 (15.4%)	43/77 (55.8%)	0.002
Peripheral eosinophilia	14/35 (40.0%)	3/5 (60.0%)	17/40 (42.5%)	0.63
Positive ANA	20/63 (31.7%)	3/14 (21.4%)	23/77 (29.9%)	0.53
Elevated Serum IgG and/or IgG4	42/46 (91.3%)	11/12 (91.7%)	53/58 (91.4%)	1.0
Elevated Serum IgG4	27/33 (81.8%)	8/10 (80.0%)	35/43 (81.4%)	1.0
Other organ involvement				
Kidney only	17 (21.5%)	3 (18.8%)	20 (21.1%)	0.58
+1 other organ	27 (34.2%)	7 (43.8%)	34 (35.8%)	
+2 other organs	19 (24.1%)	5 (31.3%)	24 (25.3%)	
+3 or more other organs	16 (20.3%)	1 (6.3%)	17 (17.9%)	
Kidney + any number of other organs	62/79 (78.5%)	13/16 (81.3%)	75/95 (78.9%)	1.0
Acute interstitial nephritis				0.21
IgG4-TIN with interstitial fibrosis	99 (94.3%)	11 (84.6%)	110 (93.2%)	
AIN	6 (5.7%)	2 (15.4%)	8 (6.8%)	
IHC IgG4				
≤10 HPF	6 (5.7%)	4 (23.5%)	10 (8.1%)	0.03
>10 HPF	100 (94.3%)	13 (76.5%)	113 (91.9%)	
≤30 HPF	34 (32.1%)	8 (50.0%)	42 (34.4%)	0.17
>30 HPF	72 (67.9%)	8 (50.0%)	80 (65.6%)	
IHC IgG4 to IgG ratio				
<0.4	31 (40.3%)	4 (44.4%)	35 (40.7%)	1.00
≥0.4	46 (59.7%)	5 (55.6%)	51 (59.3%)	
TBM deposits (IF or EM)				
No	22 (21.6%)	7 (35.0%)	29 (23.8%)	0.25
Yes	80 (78.4%)	13 (65.0%)	93 (76.2%)	
Length of follow-up (mo)				
Number of patients	51	14	65	0.38
Median	13.0	7.5	13.0	
Q1, Q3	5.0, 48.0	1.1, 52.0	4.0, 48.0	
Range	(1.0–128.0)	(0.8–120.0)	(0.8–128.0)	
Treatment, *N* patients (when known)	56	13	69	
Steroids	48 (85.7%)	11 (84.6%)	59 (85.5%)	1.0
Rituximab/Rituxan	14 (25.0%)	4 (30.8%)	18 (26.1%)	0.73
Mycophenolate mofetil	10 (17.9%)	3 (23.1%)	13 (18.8%)	0.70
Cyclophosphamide	1 (1.8%)	1 (7.7%)	2 (2.9%)	0.34
No treatment/surgery only	6 (10.7%)	1 (7.7%)	7 (10.1%)	1.0
Creatinine on follow-up (mg/dl)				
Number of patients	49	14	63	0.67
Mean (SD)	1.9 (1.1)	1.9 (1.2)	1.9 (1.1)	
Median	1.7	1.4	1.6	
Range	(0.6–7.8)	(0.7–4.9)	(0.6–7.8)	
≤1.2 (normal)	13 (25.0%)	6 (40.0%)	19 (28.4%)	0.33
>1.2 (high) or ESKD/dialysis	39 (75.0%)	9 (60.0%)	48 (71.6%)	
ESKD or dialysis on follow-up (when known)	2/52 (3.8%)	1/15 (6.7%)	3/67 (4.5%)	
Response/outcome^b^				
Nonresponse	12 (25.5%)	4 (40.0%)	16 (28.1%)	0.44
Response (creatinine decrease ≥0.3 mg/dl)	35 (74.5%)	6 (60.0%)	41 (71.9%)	

AIN, acute interstitial nephritis; ANA, antinuclear antibodies; EM, electron microscopy; ESKD, end-stage kidney disease; HPF, high power microscopic field; IF, immunofluorescence; MGN, membranous glomerulonephritis; TIN, tubulointerstitial nephritis.

a *N* (%) shown unless otherwise indicated. Frequencies not adding to column total indicate missing or nonapplicable data. Denominator shown for binary variables when not equal to column total.

b Among those for whom baseline creatinine was high and follow-up information was available.

### Clinical Characteristics of Patients with IgG4-MGN

Twenty patients with IgG4-MGN were included, 14 male and 6 female, who had a mean age at biopsy of 60.5 years (range 34–75). The median creatinine was 2.1 (range 0.7–12 mg/dl); 32% had normal creatinine. One hundred percent of the patients (18/18) with information available had proteinuria. Abnormal imaging was not a primary indication for biopsy in patients with IgG4-MGN. The median proteinuria was 8.5 gm/d (range 1.2–27); 83% (13/18) had nephrotic range proteinuria. Thirty-three percent (5/15) had hematuria. Of those IgG4-MGN patients with hematuria, 1 had concurrent IgG4-TIN, 1 had concurrent IgA nephropathy, 1 had concurrent segmental endocapillary hypercellularity in glomeruli, and the remaining 2 had IgG4-MGN only without TIN. Two patients had enlarged kidneys on radiographic studies, and 1 had a kidney mass lesion; the remaining had no radiographic abnormalities of the kidneys. Eighty-one percent (13/16) of IgG4-MGN patients had extra-renal involvement by IgG4-RD, most commonly the pancreas. Patients without extra-renal IgG4-RD had a diagnosis of IgG4-MGN based on concurrent IgG4-TIN on the kidney biopsy.

Thirteen of 20 IgG4-MGN cases (65%) had IgG4-TIN on the biopsy, including 2 with AIN. Eleven of 12 patients (92%) had increased serum IgG and/or IgG4: serum IgG4 was elevated in 8 of 10 (80%) of patients with data available, with a median value of 1210 mg/dl (range 183–2060; numeric data known for 7 IgG4-MGN patients). None had a positive ANCA; 3 of 14 patients had a positive ANA (1 also with anti-dsDNA). Two of 13 (15%) had low serum C3 or C4. One patient had a positive test for hepatitis B and a history of congenital infection.

### Microscopy Findings

#### Light Microscopy Features of IgG4-TIN

**Figure 3:**
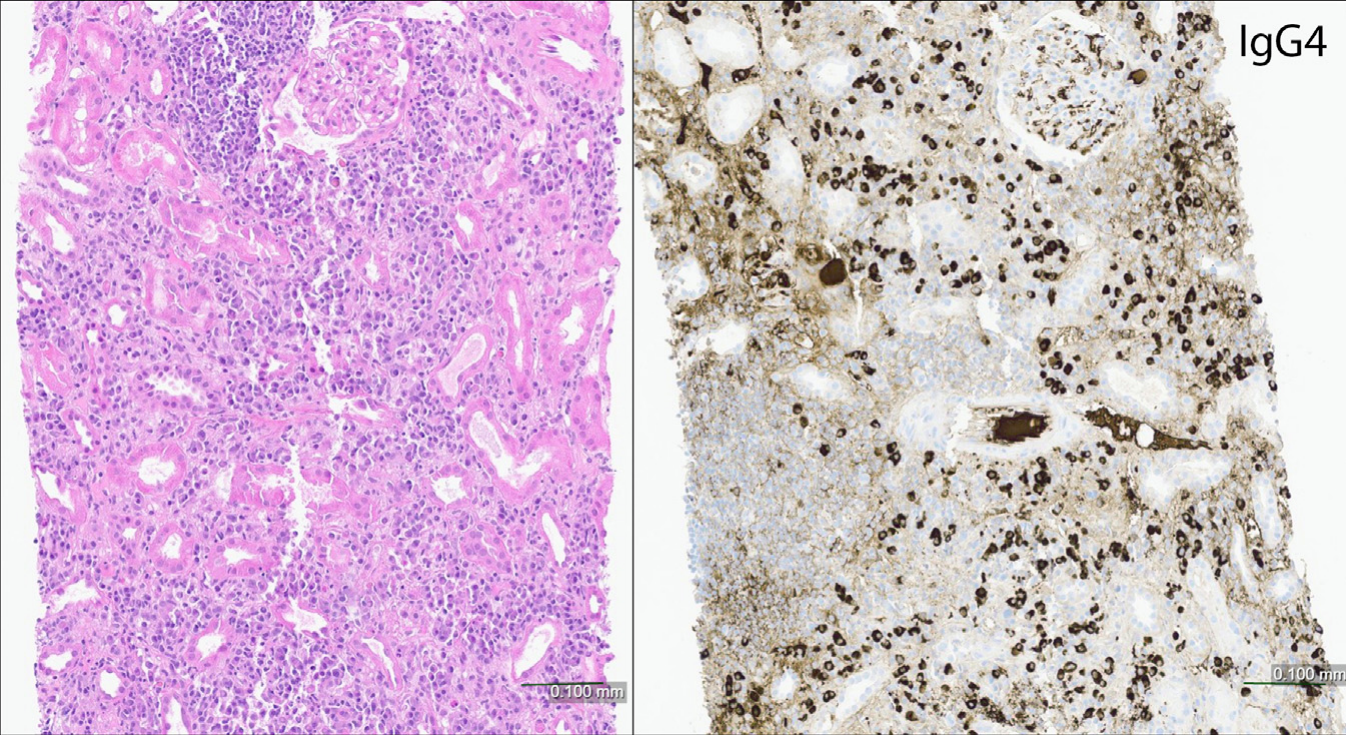
AIN pattern of IgG4-TIN. Plasma cell-rich interstitial inflammation with minimal fibrosis and no tubular atrophy (left panel). Immunoperoxidase staining for IgG4 (right panel) shows a marked increase in IgG4+ plasma cells. This pattern of injury shows minimal or no TBM immune deposits. (Hematoxylin and eosin).

**Figure 4:**
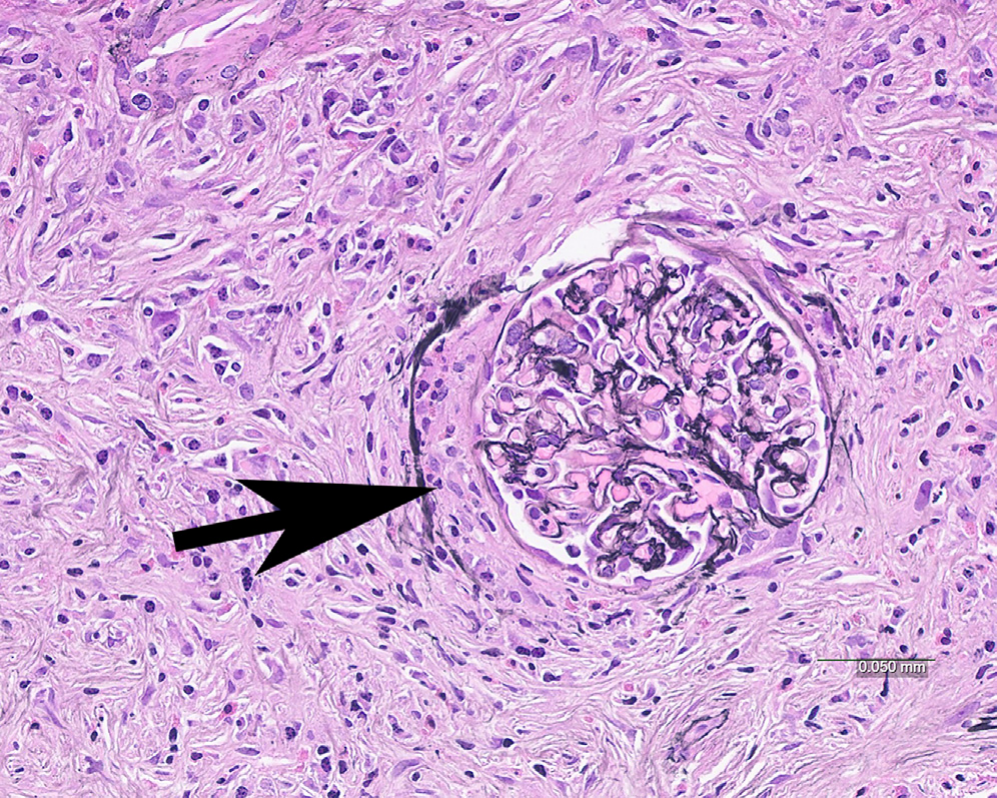
Pseudocrescent in IgG4-TIN. Interstitial fibrosis and inflammation extend into and beyond the Bowman capsule of the glomerulus causing a crescent-like pattern of injury (arrow). No fibrinoid necrosis or glomerular basement membrane disruption is present. (Jones methenamine silver).

A total of 118 kidney specimens showed IgG4-TIN, with or without concurrent MGN. In 110/118 (93%) cases of IgG4-TIN, morphologic findings by light microscopy included expansile and/or “storiform” interstitial fibrosis accompanied by mononuclear inflammatory cell infiltrate and abundant plasma cells and fewer eosinophils, and mononuclear and/or plasma cell tubulitis. Tubular atrophy accompanied interstitial fibrosis. The kidney allograft also showed chronic active antibody mediated rejection with transplant glomerulopathy, as previously reported.^18^ Of those with IgG4-TIN without IgG4-MGN, interstitial fibrosis and tubular atrophy were most frequently severe (71/108, 66%), followed by moderate (24/108, 22%). Eight patients (2 with concurrent MGN) showed an AIN pattern with minimal fibrosis (Figure 3). No granulomatous inflammation or karyorrhexis was identified in any case. Interstitial neutrophils were not prominent. Five biopsies (4%) showed “pseudocrescent” or “interstitial crescent” morphology secondary to the interstitial fibrosis and inflammation transgressing Bowman’s capsule, with resultant “capsulitis” and proliferative epithelial cells lining Bowman’s capsule, without necrosis or involvement of the glomerular tuft by a glomerulonephritis (Figure 4). Mononuclear cell tubulitis was present in all cases and was more common than plasma cell tubulitis or eosinophilic tubulitis. One biopsy showed IgG4-TIN and increased calcium oxalate crystals in tubules, thought to be due to enteric hyperoxaluria following distal pancreatectomy (for IgG4-related pancreatitis) and subsequent malabsorption. On nephrectomy samples (*n* = 5), TIN was present as a mass lesion and areas away from the mass showed no inflammation or TBM immune deposits.

**Figure 5:**
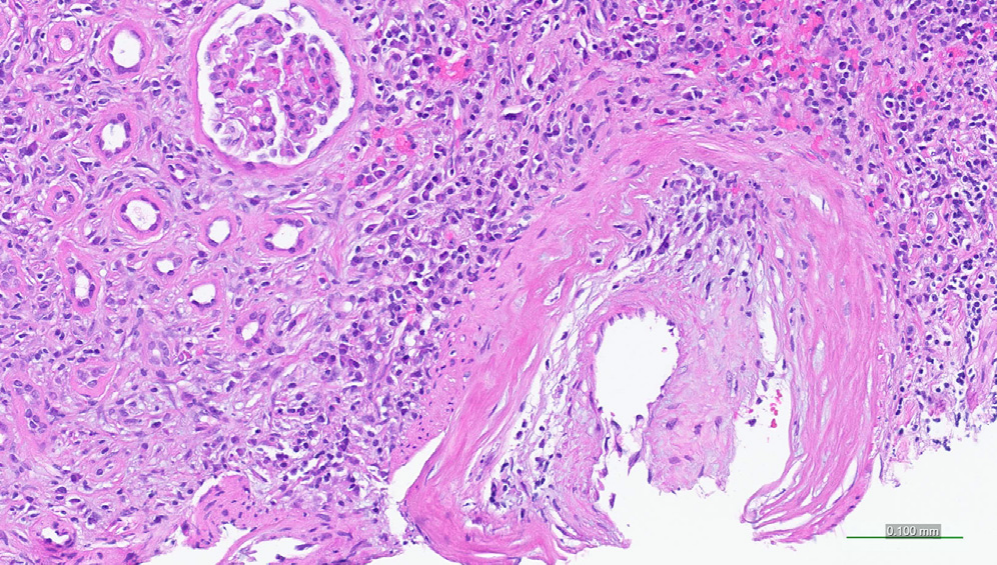
Plasma cell arteritis in IgG4-TIN. An artery shows numerous plasma cells intermixed with mononuclear cells within the thickened arterial intima. No fibrinoid necrosis is present. Note the concurrent plasma cell rich TIN pattern. Some cases of arteritis in IgG4-TIN showed mononuclear cells without significant plasma cells. (Hematoxylin and eosin).

Arteritis was present in 5 of 105 (5%) TIN cases with arteries, with biopsies showing infiltrating mononuclear cells (*n* = 3) or with a plasma cell-rich (*n* = 2) pattern (Figure 5). Arteritis involved focal arcuate or interlobular arteries and showed transmural inflammation in 2 cases and intimal inflammation in 3 cases, with associated moderate to severe fibrous intimal thickening. The arteries showed less inflammation compared with what has been reported previously.^11^ One biopsy with intimal arteritis showed an associated atheromatous embolism and severe intimal fibrosis. None of the cases with arteritis showed fibrinoid necrosis, karyorrhexis, neutrophils, giant cells, or granulomas. No inflammation in veins (“obliterative” or other phlebitis) was identified in any of the cases, including in the nephrectomy specimens.

There were 8 biopsies (7% of all IgG4-TIN specimens) that showed AIN, characterized by a plasma cell-rich inflammatory infiltrate (>10 plasma cells/HPF, and 6/8 cases with >30 plasma cells/HPF) with <10% interstitial fibrosis and tubular atrophy. These cases showed very focal (in 2 biopsies) or no TBM immune deposits by IF and/or EM. In biopsies showing the typical IgG4-TIN pattern with fibrosis, immune deposits were present in thickened TBMs in areas of interstitial fibrosis.

### Immunophenotype and Ultrastructural Features of IgG4-RKD

Of 118 IgG4-TIN cases (with or without concurrent MGN), there was at least a moderate increase (>10 cells/HPF) in IgG4+ plasma cells by immunohistochemistry in 95% of IgG4-TIN, and a marked increase (>30 cells/HPF) in 68%. The IgG4+/IgG+ plasma cell ratio was ≥0.4 in 60% (51/85) of IgG4-TIN cases. Six patients (5%) with IgG4-TIN did not show a moderate or marked increase in IgG4+ plasma cells in the tissue. Of the 6 cases with low tissue IgG4+ plasma cells, 4 of these cases had IgG immunoperoxidase staining performed and the ratio in all was <0.4. In these patients, the diagnosis of IgG4-RKD was made based on the presence of (i) typical histologic appearance (plasma cell rich TIN and moderate to severe “expansile” or “storiform” interstitial fibrosis); (ii) TBM immune deposits; and (iii) the presence of extra-renal involvement by IgG4-RD (these 3 features were present in all 6 such patients). Specifically, the extra-renal involvement in these patients included pancreas (*n* = 2), liver (*n* = 1), lung (*n* = 4), salivary gland (*n* = 1), retroperitoneal fibrosis (*n* = 1), and lymphadenopathy (*n* = 3; none of the patients had lymphadenopathy alone as extrarenal involvement). The serum IgG and/or IgG4 was elevated in all 3 patients with known values who had low tissue IgG4.

**Figure 6:**
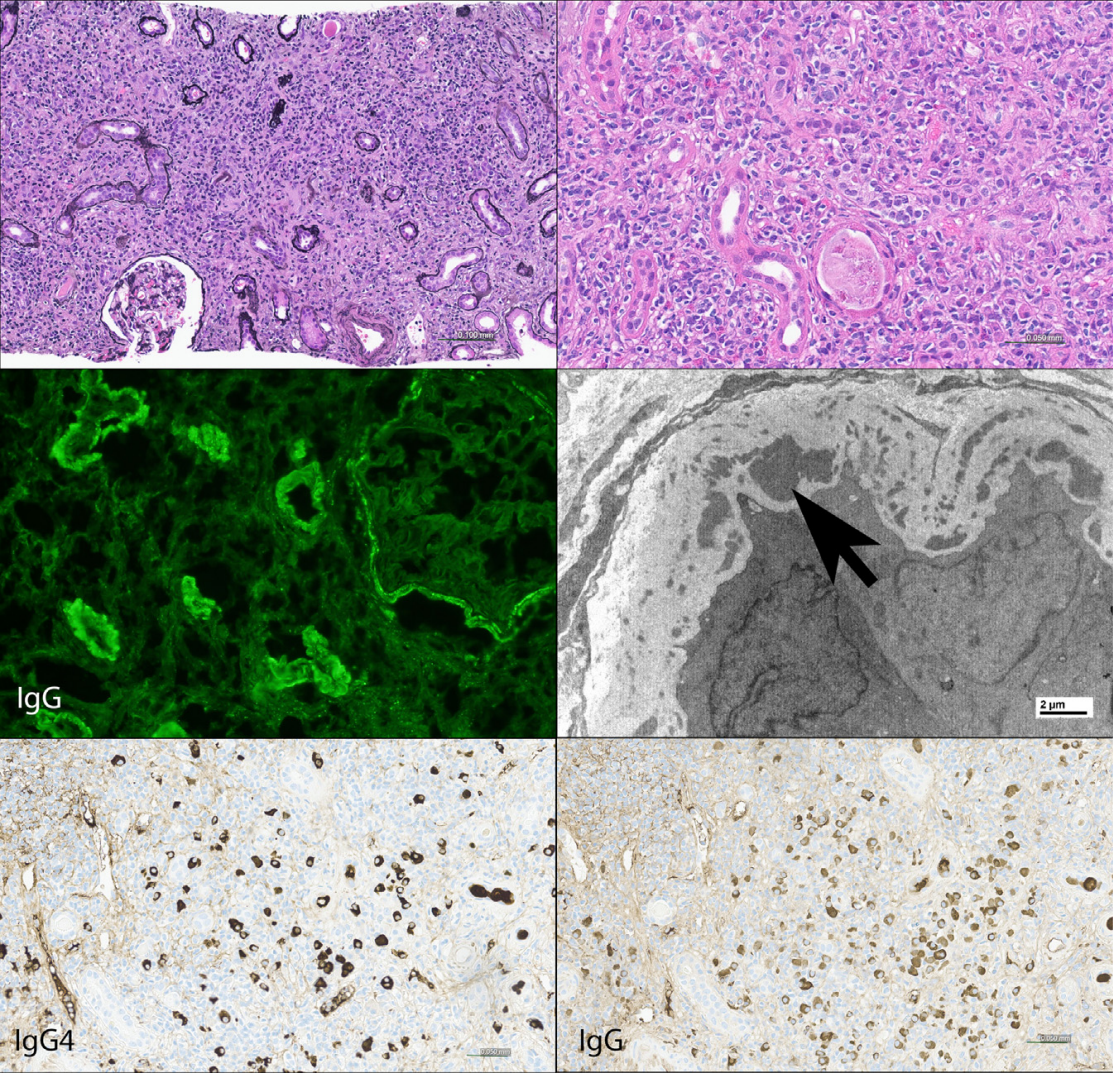
Typical appearance of IgG4-TIN. A Jones-methenamine silver stain shows an expanded interstitium by a fibroinflammatory process (upper left panel). On higher magnification, the infiltrate is composed of numerous plasma cells, mononuclear cells, and several eosinophils (H & E, upper right panel). By immunofluorescence, there is granular tubular basement membrane and Bowman's capsule staining for IgG (middle left panel). By electron microscopy, amorphous electron dense deposits (arrow) are seen within the tubular basement membrane. The two bottom panels photograph of IgG4 and IgG immunoperoxidase stains of the same specimen in the same area on the slide: this example shows a marked increase in IgG4 positive plasma cells and also numerous IgG positive plasma cells, resulting in a high IgG4+ to IgG+ plasma cell ratio.

IF staining was performed on 114 IgG4-RKD specimens (3 limited with medulla only), and EM was performed on 95 specimens. Overall, cortical TBM immune deposits were present by IF and/or EM in 77% of specimens. There was bright granular TBM staining for IgG, kappa and lambda light chains, and lesser staining for C3. TBM deposits showed staining for additional immunoreactants in 55% of the cases (48/88) including C1q (36/88, 41%), IgM (26/88, 30%), and IgA (9/88, 10%). EM showed amorphous electron dense immune type deposits within the TBMs and/or interstitium. Biopsies from patients with the AIN pattern did not show significant TBM immune deposits. Typical light microscopy, IF, EM, and immunohistochemistry (IgG4 and IgG) in IgG4-TIN are shown in Figure 6.

### IgG4-MGN

The cohort included 20 cases of MGN, defined as regularly spaced subepithelial deposits along the glomerular basement membranes. Seven of 20 MGN cases (35%) showed MGN only without concurrent IgG4-TIN; in these cases, the diagnosis of IgG4-MGN was established because of the presence of extrarenal involvement by IgG4-RD. Two biopsies with MGN and TIN showed an AIN pattern. One biopsy showed a segmental MGN pattern. One biopsy showed a global MGN pattern along with endocapillary hypercellularity and small mesangial and subendothelial deposits.

In the glomeruli, IF studies showed 1 to 3+ granular global glomerular basement membranes staining for IgG, C3, and kappa and lambda light chains in 18; 1 biopsy showed 1+ staining for IgG, C3, and kappa with negative staining for lambda; 1 biopsy showed segmental mesangial and glomerular basement membranes dim staining for C3 only by IF and showed a segmental membranous pattern by EM. Eight cases showed mesangial deposits by IF in addition to the membranous pattern. In some cases, there was additional staining in a membranous pattern by IF with lesser intensity for C1q (*n* = 8), IgA (*n* = 7), and IgM (*n* = 7). PLA2R staining was negative in 11 of 12 stained biopsies. Thrombospondin type-1 domain–containing 7A was further stained in 2 of the PLA2R–negative cases and was also negative. TBM immune deposits were seen in 60% (12/20) of the MGN cases, by IF or EM. One biopsy with MGN showed an additional component of IgA nephropathy.

EM showed subepithelial amorphous electron dense deposits along the glomerular basement membranes in all cases, subendothelial deposits in 5 cases, mesangial deposits in 9 cases, and endothelial tubuloreticular inclusions in 5 cases. One biopsy showed a focal microspherical substructure of the subepithelial deposits.

### Other Glomerulopathies in IgG4-RKD

In addition to IgG4-MGN, in our cohort we found glomerular disease as follows: diabetic glomerulosclerosis (*n* = 14; 12% of biopsies), IgA nephropathy (*n* = 3; 2.5% of biopsies), and mild mesangial proliferative immune complex glomerulonephritis (*n* = 8; 6.6% of biopsies).

### Histologic, Immunophenotypic, and Laboratory Correlations

We evaluated biopsy/nephrectomy features and corresponding laboratory features, with particular emphasis on serum IgG and/or IgG4 and hypocomplementemia. Patients with IgG4-TIN alone were more likely to have hypocomplementemia than those with IgG4-MGN (+/− TIN) (*P* = 0.002). Additional comparisons with hypocomplementemia were performed among patients with TIN. Hypocomplementemia was associated with elevated total serum IgG (*P* = 0.05), but not with elevated serum IgG4. Hypocomplementemia was associated with the severity of interstitial fibrosis and tubular atrophy (76% of patients with hypocomplementemia had severe interstitial fibrosis and tubular atrophy (IFTA), as compared with only 45% of nonhypocomplementemic patients having severe IFTA, *P* = 0.02). Although patients with hypocomplementemia were slightly more likely to have TBM deposits than those without hypocomplementemia (83% vs. 70%), this was not statistically significant (*P* = 0.25). Hypocomplementemia was not significantly associated with other organ involvement (presence or absence, or number of organs involved), peripheral eosinophilia, or tissue IgG4 plasma cell concentration in patients with IgG4-TIN. Patients with hypocomplementemia were more likely to have a positive ANA than those without hypocomplementemia (50% vs. 14%, *P* = 0.006). There was no significant association between tissue IgG4 plasma cell concentration and serum IgG4 level, although elevated tissue and/or serum IgG4 were part of the diagnostic criteria for IgG4-RD and thus prevalent in our cohort. Among 34 patients with elevated serum IgG4, 29 (85%) had >10 IgG4+ cells/HPF, as compared with 100% of 8 patients with nonelevated serum with >10 cells/HPF (*P* = 0.56). Further, 68% and 50% of patients with IgG4 elevated and nonelevated serum (respectively) had >30 IgG4+ cells/HPF (*P* = 0.42).

### Patient Follow-up

Follow-up clinical information (with known creatinine, ESKD/dialysis status, or clinical note) was available in 74 patients, at a median of 13 months (range 0.8–128 months) after biopsy or nephrectomy (*N* = 65 patients with known follow-up date). Fifty-nine of 69 patients (86%) were treated with steroids (prednisone or prednisolone), with or without additional immunosuppressive treatment. 18/69 patients (26%) were treated with rituximab, 13/69 (19%) with mycophenolate mofetil, and 2 (3%) each with azathioprine or cyclophosphamide. The median baseline creatinine among all patients was 2.5 mg/dl (range 0.7–12 mg/dl) at the time of biopsy/nephrectomy and in follow up (*N* = 63) was 1.6 mg/dl (range 0.6–7.8 mg/dl). Among patients with known creatinine at baseline and follow-up, creatinine decreased by a median of 0.7 mg/dl (interquartile range −1.9 to −0). Of those patients with elevated creatinine at baseline and known response status, 72% (41/57) showed a response (defined as decrease in creatinine of ≥0.3 mg/dl). Four patients developed ESKD. Among the 8 patients with IgG4-TIN with an AIN pattern of injury, follow-up was available in 6 patients, and all (100%) showed improvement of their kidney function as compared to 69% of non-AIN pattern patients showing response (*P* = 0.17). The following biopsy features were not associated with treatment response: interstitial fibrosis and tubular atrophy (designated as none, mild, moderate, or severe; or dichotomized as none/mild or moderate/severe), presence of TBM immune deposits (by IF or EM), arteritis, or tissue IgG4 (designated as no or mild increase, moderate, or marked increase). The following laboratory and clinical features were not associated with treatment response: elevated serum IgG and/or IgG4, hypocomplementemia, peripheral eosinophilia, positive ANA, and other organ involvement (presence or absence, and number of organs involved).

Specifically analyzing the 20 patients with IgG4-MGN, follow-up data were available in 14 patients. The median follow-up time was 7.5 months (range 0.8–120). One patient with ESKD at presentation did not receive treatment other than later kidney transplant. Eleven patients were treated with prednisone (85%), 5 with rituximab (31%), 3 with mycophenolate mofetil (23%), and 2 with cyclophosphamide. One patient also received hydroxychloroquine for a considered diagnosis of systemic lupus (with MGN and focal IgG4-TIN). Six of 10 patients (60%) showed improved serum creatinine (among those with baseline elevated creatinine), 1 had ESKD without improvement, and the remaining showed stable kidney function. Follow up information, including the degree of proteinuria, was available in 9 patients (not including the 1 patient with ESKD). All had improved proteinuria: 5 obtained complete remission of proteinuria, and 4 obtained partial remission of proteinuria (estimated proteinuria ranging from 0.6–2.4 g/d).

## Discussion

This study analyzes 125 patients with kidney tissue specimens showing IgG4-RKD, and further elucidates the histologic and immunophenotypic features and clinical and laboratory correlates in IgG4-RKD. The most common primary indication for undergoing biopsy or nephrectomy was renal failure, followed by proteinuria and abnormal imaging findings. Although not necessarily the primary reason for biopsy, mass lesion(s) or other abnormal imaging findings were present in 52% of all patients with IgG4-RKD; it is of particular interest that so many of these patients had abnormal kidney imaging studies since our center receives a relatively high volume of medical kidney biopsies compared with kidney tumor biopsies.

IgG4-TIN is commonly characterized by a plasma cell–rich interstitial nephritis with moderate-to-severe interstitial fibrosis and tubular atrophy by light microscopy, along with granular TBM immune deposits. A subset of cases shows an AIN pattern, which has not previously been well-recognized. The AIN pattern is also a plasma cell–rich TIN but is more difficult to recognize as part of IgG4-RD in that it does not show the “storiform” fibrosis pattern by light microscopy (typical of other organs involved by IgG4-RD), and TBM deposits are absent or minimal. Although it was a small number of cases, this AIN subset showed a 100% response rate to treatment. Because of the difficulty in diagnosis, we suggest that immunoperoxidase staining for IgG4 be performed in all cases of plasma cell rich TIN. Pathologists must recognize that other diseases may also show at least focal increased IgG4+ plasma cells.^10^^,^^21^ In particular, ANCA associated disease can show a diffuse marked increase in IgG4-positive plasma cells, and can be mass-forming in the kidney and in other organs.^21^ The differential diagnosis includes other types of plasma cell–rich interstitial inflammation, especially if cases show focal increased IgG4-positive plasma cells. Some of these entities may show radiographic abnormalities, such as chronic pyelonephritis, idiopathic multicentric Castleman disease, Sjogren syndrome (some case reports of mass-forming Sjogren syndrome likely represent IgG4-TIN, however), and extramedullary hematopoiesis.^22^, ^23^, ^24^ Other types of plasma cell–rich interstitial nephritis without radiographic abnormalities include antibrush border antibody disease (some cases of which show increased IgG4-positive plasma cells), tubulointerstitial nephritis with uveitis, IgM plasma cell interstitial nephritis, and other types of autoimmune interstitial nephritis as well as infectious interstitial nephritis (reviewed by Gilani *et al.*).^25^, ^26^, ^27^, ^28^ Because none of our IgG4-TIN cases showed granulomatous inflammation, the presence of any type of granulomatous inflammation argues strongly against IgG4-TIN.

Although immunoperoxidase staining for IgG4 is useful in evaluating IgG4-TIN, we found that 5% of IgG4-TIN cases actually did not have an increase in IgG4+ plasma cells. In these patients, the diagnosis can be established by a typical histologic appearance, presence of TBM immune complex deposits, and other organ involvement by IgG4-RD. An increased IgG4+ to IgG+ plasma cell ratio (defined as >0.4 in other organs) has been proposed as a diagnostic tool, particularly for tissues involved by IgG4-RD that show less inflammation and more fibrosis (e.g., retroperitoneal fibrosis).^19^ However, we found that an increased IgG4+ to IgG+ plasma cell ratio was present in only 60% of IgG4-TIN, and so this is not a helpful diagnostic feature. These findings reinforce that IgG4 is a biomarker, rather than a unique defining feature, of IgG4-RKD.

Another histologic feature we observed in IgG4-RKD is small vessel arteritis. Arteritis as a part of IgG4-RD has been well described in medium-sized and large vessels.^29^^,^^30^ Small vessel arteritis, as reported in this series, has not been as well recognized in IgG4-RD, either in the kidney or in other organs. Arteritis in the kidney was first described as a case report of IgG4 plasma cell arteritis in 2013 by Sharma *et al.*^11^ In our series, arteritis (plasma cell–rich or with only mononuclear cells) was uncommon. However, the presence of small vessel arteritis in the kidney expands the spectrum of IgG4-related arteritis, and argues for inclusion of IgG4-related arteritis into the category of “variable vessel vaculitis”, which can affect vessels of any size.^31^ The absence of fibrinoid necrosis, neutrophils, and karyorrhexis in arteries helps to distinguish IgG4-related arteritis from ANCA-associated disease, which also can show increased IgG4+ plasma cells in the interstitium.^21^ “Pseudocrescents” were also rarely present but are a distinctive feature of IgG4-TIN and should not be confused for a glomerulonephritis (particularly pauci-immune glomerulonephritis). Also of note, we did not observe “obliterative phlebitis,” as has been considered a characteristic histologic feature in other organs affected by IgG4-RD, or other inflammation in veins in the kidney.^19^ One potential explanation for the lack of phlebitis in the kidney may be the relative lack of muscularis in intrarenal veins compared to veins in other organs.^32^

This study also shows the range of glomerulonephritis in IgG4-RKD. This series contains the largest number of IgG4-MGN (20 cases), which were analyzed separately. Except for 1 case, none were associated with PLA2R, arguing that this pattern of injury is a not a primary membranous nephropathy. Furthermore, several cases showed other features suggestive of a secondary MGN, including mesangial deposits and/or tubuloreticular inclusions in endothelial cells. IgG4-MGN can occur with or without associated TIN; if not co-occuring with IgG4-TIN, then the diagnosis must be made based on other organ involvement by IgG4-RD. Other glomerular diseases we observed were diabetic glomerulosclerosis (in 12% of biopsies), IgA nephropathy, and immune complex glomerulonephritis not otherwise specified. Although diabetic glomerulosclerosis is common in the general population, it may be a part of IgG4-RD when this disease involves the pancreas and affects endocrine function.^33^

We found that the kidney was the only organ involved by IgG4-RD in 21% of our cases. Therefore, the pathologist and nephrologist should be alert to the possibility of establishing the diagnosis of IgG4-RD with kidney-only involvement, at least at the time of presentation of kidney disease. Because IgG4-RD is a systemic disease and often requires a multidisciplinary approach, the nephrologist may also refer the patient for further assessment (such as clinical and radiologic evaluation) to evaluate the extent of the disease that may not otherwise have been recognized.

Compared with what has been reported in the literature overall in IgG4-RD, we found that patients with kidney involvement by IgG4-RD more frequently have hypocomplementemia and increased serum IgG4 or IgG.^1^^,^^34^^,^^35^ It has been suggested that the combination of renal involvement, hypocomplementemia, and increased serum IgG4 portends a more severe phenotype of IgG4-RD.^1^^,^^36^ In this study, we found an association of hypocomplementemia with more severe interstitial fibrosis and tubular atrophy, and increased serum total IgG; these findings may indicate a more severe phenotype of IgG4-RKD, although hypocomplementemia was not associated with a lack of response to treatment. Also of note, in IgG4-RKD, ANA positivity was associated with hypocomplementemia, which may lead to confusion with lupus nephritis clinically.

IgG4-RD overall, including IgG4-TIN, tends to show a high rate of response to immunosuppressive therapy, although there can be a high relapse rate following discontinuation of immunosuppression.^10^^,^^37^ In this study, which was limited due to lack of consistent clinical and laboratory follow-up data on all patients, we found a slightly lower response to therapy at last follow-up than what has been reported previously. However, even in patients with moderate-to-severe interstitial fibrosis and tubular atrophy on biopsy, there was a response to therapy, possibly because the disease with fibrosis shows patchy involvement of the kidney and a biopsy is not representative of the kidney overall, or possibly because such fibrosis behaves differently from fibrosis in other disease processes. Therefore, nephrologists should not dismiss the option of treatment with immunosuppression in patients with severe fibrosis on biopsy.

In summary, this is the largest tissue-based series of IgG4-RKD. With this study, we elucidate the clinicopathologic and laboratory characteristics of the renal disease, expand on the subsets characterized by IgG4-MGN and AIN components, confirm the presence of small artery involvement in a subset of cases, and recognize other glomerulopathies that are seen in IgG4-RD. It is important to be aware of the multiple pathological features of IgG4-RKD to allow for accurate diagnosis, systemic evaluation of patients, and appropriate treatment.

## Disclosure

The manuscript was not prepared by or funded by a commercial organization. LDC reports serving on an advisory board for Amgen, Inc. All the authors declared no competing interests.

## Data Availability Statement

All relevant deidentified data supporting the findings of this study are available from the corresponding author upon reasonable request, after approval from the institutional review board.

## Supplementary Material

Supplementary File (PDF) **STROBE Statement** (PDF).
