# Supplementary material for: Clinicopathologic Features of IgG4-Related Kidney Disease
Source: Kidney Int Rep. 2024 May 15;9(8):2462–73. doi: 10.1016/j.ekir.2024.05.011 (PMC11328570; doi:10.1016/j.ekir.2024.05.011)
Supplement: Supplementary File (PDF) — STROBE Statement (PDF). [file mmc1.pdf]

STROBE Statement—checklist of items that should be included in reports of observational studies

	Item No.	Recommendation	Page No.	Relevant text from manuscript
**Title and abstract**	1	(*a*) Indicate the study's design with a commonly used term in the title or the abstract	2	"We conducted a tissue-based cohort study with clinicopathological correlation…"
		(*b*) Provide in the abstract an informative and balanced summary of what was done and what was found	2	
**Introduction**				
Background/rationale	2	Explain the scientific background and rationale for the investigation being reported	3	
Objectives	3	State specific objectives, including any prespecified hypotheses	4	"The goal of our study is to enrich the current clinicopathological knowledge of IgG4-RKD… in this largest tissue-based series of IgG4-RKD."
**Methods**				
Study design	4	Present key elements of study design early in the paper	4-5	
Setting	5	Describe the setting, locations, and relevant dates, including periods of recruitment, exposure, follow-up, and data collection	4-7	"126 patients consecutively diagnosed with IgG4-RKD between January 2001 and April 2023", "Biopsies and nephrectomies were obtained from patients who underwent biopsy or nephrectomy at Mayo Clinic, from patients who underwent medical renal biopsy outside of Mayo Clinic and had

				tissue sent to Mayo Clinic for processing, and a small number of consult cases initially interpreted by pathologists elsewhere and reviewed at the Mayo Clinic in Rochester, Minnesota.", "Presentation and medical history, imaging, laboratory findings, treatment, and follow-up were obtained from referral forms submitted at the time of biopsy and patients' medical records."
Participants	6	(*a*) *Cohort study*—Give the eligibility criteria, and the sources and methods of selection of participants. Describe methods of follow-up	4-5	"To qualify as IgG4-TIN, renal specimens must have shown a plasma cell-rich tubulointerstitial nephritis (TIN) pattern and at least one of the following criteria: (1) Clinical evidence of other organ involvement by IgG4-RD, (2) Laboratory results of increased serum total IgG or IgG4 levels or hypergammaglobulinemia, or (3) Radiographic features in the kidney…", "To qualify as IgG4-MGN, renal biopsies must have shown a membranous glomerulonephritis pattern of injury and concurrent IgG4-TIN or other organ involvement by IgG4-RD."

		(*b*) *Cohort study*—For matched studies, give matching criteria and number of exposed and unexposed	NA	
Variables	7	Clearly define all outcomes, exposures, predictors, potential confounders, and effect modifiers. Give diagnostic criteria, if applicable	4-5	"To qualify as IgG4-TIN, renal specimens must have shown a plasma cell-rich tubulointerstitial nephritis (TIN) pattern and at least one of the following criteria: (1) Clinical evidence of other organ involvement by IgG4-RD, (2) Laboratory results of increased serum total IgG or IgG4 levels or hypergammaglobulinemia, or (3) Radiographic features in the kidney…", "To qualify as IgG4-MGN, renal biopsies must have shown a membranous glomerulonephritis pattern of injury and concurrent IgG4-TIN or other organ involvement by IgG4-RD.", "Laboratory data included: serum creatinine, serum protein electrophoresis, … Among those with elevated creatinine at the time of biopsy/nephrectomy, we defined treatment response as a decrease of at least 0.3 mg/dL in creatinine. Patients with end-stage kidney disease (ESKD) or dialysis on follow-up were

				included among treatment non-responders.”
Data sources/ measurement	8*	For each variable of interest, give sources of data and details of methods of assessment (measurement). Describe comparability of assessment methods if there is more than one group	6	
Bias	9	Describe any efforts to address potential sources of bias	6-7	
Study size	10	Explain how the study size was arrived at	4	“…patients consecutively diagnosed with IgG4-RKD between January 2001 and April 2023”

Continued on next page

Quantitative variables	11	Explain how quantitative variables were handled in the analyses. If applicable, describe which groupings were chosen and why	5-7
Statistical methods	12	(*a*) Describe all statistical methods, including those used to control for confounding	7
		(*b*) Describe any methods used to examine subgroups and interactions	5,7
		(*c*) Explain how missing data were addressed	7
		(*d*) *Cohort study*—If applicable, explain how loss to follow-up was addressed<br>*Case-control study*—If applicable, explain how matching of cases and controls was addressed<br>*Cross-sectional study*—If applicable, describe analytical methods taking account of sampling strategy	NA
		(*e*) Describe any sensitivity analyses	NA
**Results**			
Participants	13*	(a) Report numbers of individuals at each stage of study—eg numbers potentially eligible, examined for eligibility, confirmed eligible, included in the study, completing follow-up, and analysed	7
		(b) Give reasons for non-participation at each stage	
		(c) Consider use of a flow diagram	
Descriptive data	14*	(a) Give characteristics of study participants (eg demographic, clinical, social) and information on exposures and potential confounders	NA
		(b) Indicate number of participants with missing data for each variable of interest	Table 1
		(c) *Cohort study*—Summarise follow-up time (eg, average and total amount)	
Outcome data	15*	*Cohort study*—Report numbers of outcome events or summary measures over time	7-8
		Case-control study—Report numbers in each exposure category, or summary measures of exposure	
		Cross-sectional study—Report numbers of outcome events or summary measures	
Main results	16	(*a*) Give unadjusted estimates and, if applicable, confounder-adjusted estimates and their precision (eg, 95% confidence interval). Make clear which confounders were adjusted for and why they were included	7-10, 15-16
		(*b*) Report category boundaries when continuous variables were categorized	7-10, 15-16
		(*c*) If relevant, consider translating estimates of relative risk into absolute risk for a meaningful time period	NA

Continued on next page

Other analyses	17	Report other analyses done—eg analyses of subgroups and interactions, and sensitivity analyses	7-14	
**Discussion**				
Key results	18	Summarise key results with reference to study objectives	16-17	
Limitations	19	Discuss limitations of the study, taking into account sources of potential bias or imprecision. Discuss both direction and magnitude of any potential bias	19	"limited due to lack of consistent follow-up data on all patients"
Interpretation	20	Give a cautious overall interpretation of results considering objectives, limitations, multiplicity of analyses, results from similar studies, and other relevant evidence	19-20	
Generalisability	21	Discuss the generalisability (external validity) of the study results	20	
**Other information**				
Funding	22	Give the source of funding and the role of the funders for the present study and, if applicable, for the original study on which the present article is based	20	

*Give information separately for cases and controls in case-control studies and, if applicable, for exposed and unexposed groups in cohort and cross-sectional studies.

Note: An Explanation and Elaboration article discusses each checklist item and gives methodological background and published examples of transparent reporting. The STROBE checklist is best used in conjunction with this article (freely available on the Web sites of PLoS Medicine at http://www.plosmedicine.org/, Annals of Internal Medicine at http://www.annals.org/, and Epidemiology at http://www.epidem.com/). Information on the STROBE Initiative is available at www.strobe-statement.org.
